# Enhancement of BMP-2 and VEGF carried by mineralized collagen for mandibular bone regeneration

**DOI:** 10.1093/rb/rbaa022

**Published:** 2020-06-13

**Authors:** Kun Liu, Chun-Xiu Meng, Zhao-Yong Lv, Yu-Jue Zhang, Jun Li, Ke-Yi Li, Feng-Zhen Liu, Bin Zhang, Fu-Zhai Cui

**Affiliations:** 1 Shandong Provincial Key Laboratory of Oral Tissue Regeneration, Shandong Engineering Laboratory for Dental Materials and Oral Tissue Regeneration, School and Hospital of Stomatology, Shandong University, Jinan, Shandong 250012, P. R. China; 2 Liaocheng People’s Hospital, Medical College of Liaocheng University, Liaocheng 252000, P. R. China; 3 College of Materials Science and Engineering of Liaocheng University, Liaocheng 252000, P. R. China; 4 Department of Materials Science and Engineering, Tsinghua University, Beijing 100084, P. R. China

**Keywords:** bone regeneration, mineralized collagen, BMP-2, VEGF

## Abstract

Repairing damage in the craniofacial skeleton is challenging. Craniofacial bones require intramembranous ossification to generate tissue-engineered bone grafts via angiogenesis and osteogenesis. Here, we designed a mineralized collagen delivery system for BMP-2 and vascular endothelial growth factor (VEGF) for implantation into animal models of mandibular defects. BMP-2/VEGF were mixed with mineralized collagen which was implanted into the rabbit mandibular. Animals were divided into (i) controls with no growth factors; (ii) BMP-2 alone; or (iii) BMP-2 and VEGF combined. CT and hisomputed tomography and histological staining were performed to assess bone repair. New bone formation was higher in BMP-2 and BMP-2-VEGF groups in which angiogenesis and osteogenesis were enhanced. This highlights the use of mineralized collagen with BMP-2/VEGF as an effective alternative for bone regeneration.

## Introduction

Segmental mandibular defects caused by tumor resection, genetic disorders or trauma remains challenging [1–3]. Unrepaired defects are associated with defacement, reduced masticatory capability and loss of speech, which severely affect the patient’s quality of life. Autologous bone grafts are the first-line treatment for segmental mandibular reconstruction [4, 5] which can be improved by tissue engineering approaches [3, 6]. In the light of this, tissue engineering might offer a next step in the evolution of mandibular reconstruction. The scaffolds are one of critical factors for tissue engineering and regeneration. In modern medicine, bio-scaffolds from bioceramics and polymers components that improve bone growth are of particular interest. In particular, scaffolds that recapitulate the molecular cues of bone healing are currently under development. Several biomaterials have been developed to control and sustain the delivery of BMP-2. Scaffolds such as hyaluronic acid-based hydrogel supported bone growth with BMP-2 known to enhance bone formation [7]. Currently, much experimentation has focused on the efficacy of BMP-2 for other applications but less for segmental mandible defects [8]. The use of BMP-2 is, however, hindered by the requirement for high effective doses leading to side-effects such as swelling, seroma formation and cystic bone lesions. To prevent such complications, more effective BMP-2 delivery methods are required. Vascular endothelial growth factor (VEGF) improves the transport of precursor mesenchymal cells to the mineralized regions of bone through angiogenesis [9, 10]. The use of VEGF with low dose BMP-2 may, therefore, act in concert to promote bone healing, particularly for the early functional loading of dental implants following bone grafting. The challenge is the ability to engineer materials that match the biological and mechanical properties of the real bone tissue matrix that maintain the ability to support bone vascularization.

However, controversy remains whether co-treatment with VEGF/BMP-2 produces beneficial effects in comparison to BMP-2 alone, particularly at the mandibular bone that possesses an abundant vasculature. In this study, we assessed the effects of VEGF combined with BMP-2 through mineralized collagen delivery into mandibular alveolar bone defects in rabbits. The effectiveness of VEGF-BMP2 co-delivery was compared with BMP-2 alone.

## Methods

### Scaffold fabrication

Mineralized Collagen was purchased from Beijing Allgens Medical Science [11–13]. Mineralized collagen scaffolds were prepared through the formation of mineralized type I collagen fibrils that were synthesized through self-assembling hydroxyapatite (HA) crystals that grew within the collagen fibrils and to form triple collagen self-assembled helices. During mineralization, HA growth and nucleation were regulated by the collagen fibrils in a manner comparable to *in vivo* bone mineralization. The resultant solutions were freeze-dried to produce MC, which was shaped and sterilized though 60 Co irradiation.

### Surgical assessments

**Figure 1:**
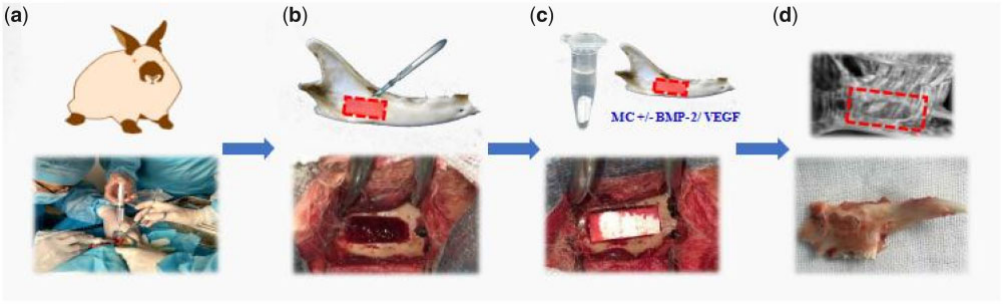
Application of scaffolding to mandibular alveolar bone defects. (**a**) Experimental procedures, (**b**) mandibular alveolar bone defect (10 × 4×2 mm^3^) created, (**c**) application of mineralized collagen and growth factor complex, (**d**) after 12 weeks, the growth state of the mandible sample.

All animal protocols were approved by our institutional review board and followed the guidelines of the Declaration of Helsinki. New Zealand white rabbits aged 11–12 weeks (200–300 g) were assessed. Rabbits were housed in a natural light/dark cycle of 12 h at 15–21°C and provided free access to food and water. Rabbits were anesthetized through injection with pentobarbital sodium (0.038 mg/g) and xylazine hydrochloride (0.075 mg/g). Submandibular incisions were produced in the skin, subcutaneous tissue and masseter muscles, parallel to the inferior border of the left mandible. Lingual and buccal surfaces were then exposed to an elevator, and 10 × 4×2 mm^3^ full-thickness defects were produced in the left mandible body using a dental drill. Saline was added dropwise to the defect to prevent overheating. Mandible bone fragments, coagulation scabs and tissues were then washed away with saline and following wound washing and hemostasis, the defects were then filled with MC alone or MC/BMP-2 (BMP-2: 100 µg/ml) or MC/BMP-2/VEGF (BMP-2: 100 µg/ml; VEGF: 10 µg/ml), the subcutaneous fascia, skin and muscle incisions were sutured with absorbable 4/0 sutures following saline irrigation and alcohol/iodine sterilization (Fig. 1). Post-surgery, rabbits were injected into the muscle with penicillin for three successive days. Rabbits were independently housed over the postoperative period and irregular behavior and operative complications were monitored. Rabbits were provided free access to food and water during the recovery period.

### CT assessments

Mandibles of 9 sacrificed animals were assessed at 4, 8 and 12 weeks, respectively. Untreated left-sided defects were compared. Mandible morphology was imaged via CT on a Toshiba Aquilion 16 under the indicated parameters: 120 kV, 125 mA, 1-s scan and 0.5 mm sections. Mandibles were imaged in pseudo 3D displays.

### CT analysis

A Leica Qwin Pro image analyzer was used to assess bone ingrowth. Image analysis was performed on the surface area and surrounding bone. Data are shown as arbitrary units and reflect the levels of bone filling. Bone ingrowth was calculated at different times post-surgery.

### *In vivo* histology

Bone was obtained at weeks 4, 8 and 12 to assess the infiltration of the implants. Bone sections of 900 nm were prepared using a rotating diamond wafering saw. Sections were mounted onto slides, ground to ≤100 mm, and polished. Sections were H&E stained using Kossa silver nitrate, and counterstained with safranin (Sigma) as per the manufacturer’s recommendations.

### IHC 

Samples obtained from the implanted bone regions were fixed at week 12 in 4% PFA prior to paraffin embedding. To assess human vimentin expression using Immunohistochemistry (IHC), endogenous peroxidase was quenched through incubation with 1% H_2_O_2_ and methanol. Paraffin sections were labeled with human anti-vimentin antibodies and developed using hematoxylin.

## Results

### Clinical assessments

All animals displayed normal behavioral patterns and healing, with minimal inflammation observed. Upon analysis, each of the wounds closed over the 12-week healing period. Post-implantation, two rabbits were sacrificed and the bony mandibles were dissected and analyzed. The formation of bone could be observed macroscopically at the implanted regions.

### CT images

**Figure 2:**
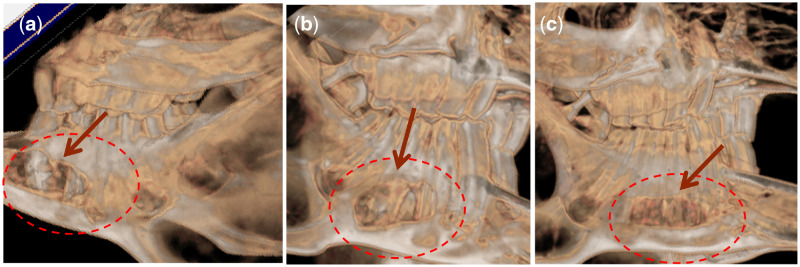
CT Images of mandibular alveolar bone defects implanted with (a) MC, (b) MC/BMP-2, (c) MC/BMP-2/VEGF after 12 weeks.

To investigate bone unions in the defects, CT images were obtained at the indicated timepoints up to 12 weeks post-operatively (Fig. 2). Cell-scaffolds were observed on the right mandible side 4 weeks post-operatively. By 8–12 weeks, the defects were comparable to native bone, but delineation of the margins of the defects was not possible.

### CT analysis

**Figure 3:**
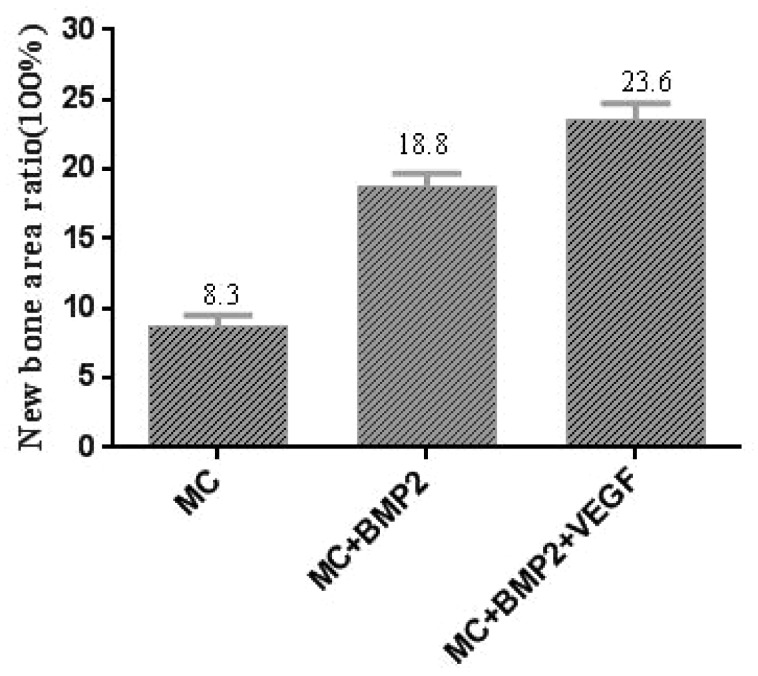
Histomorphometric analysis of newly formed bone.

**Figure 4:**
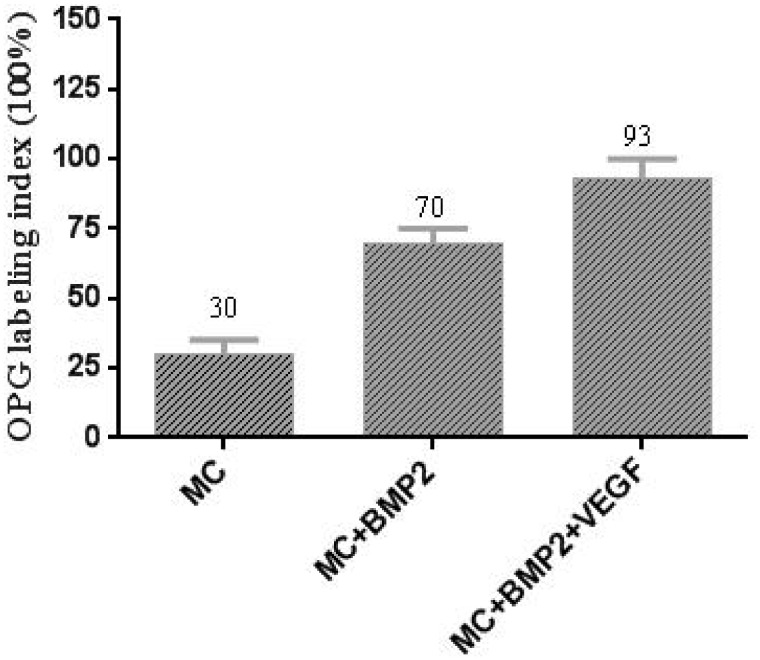
Immunohistochemical analysis of OPG positive cells staining ratio.

The CT analysis showed that the density of the defects filled with the designed osteoblasts-scaffolds were higher than those that were unfilled. Data were acquired at weeks 4, 8 and 12 post-scaffold implantation (Figs 3 and 4). After 12 weeks, bone formation in the implanted areas exceeded that of the 4-week group. Significant differences were also evident from 4 to 12 weeks.

The ratio of new bone was calculated using the following equation:

$$\text{New}\;\text{bone}\;\text{area}\;\text{ratio}\;\left(\%\right)=\left(\frac{\text{newly}\;\text{formed}\;\text{bone}\;\text{area}\;(mm^{2})}{\text{total}\;\text{area}\;(mm^{2})}\right)\;*\;100\left(％\right).$$

### *In vivo* histology

**Figure 5:**
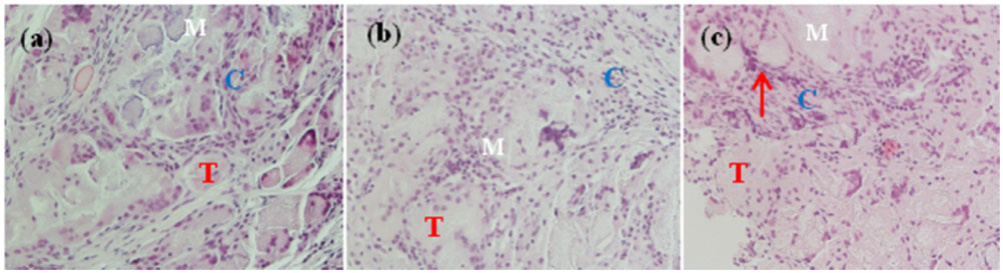
Hematoxylin–eosin photographs (H&E ×20 magnification) of mandibular alveolar bone defects implanted with (**a**) MC, (**b**) MC/BMP2 and (**c**) MC/BMP2/VEGF at 12 weeks after surgery. C: osteoblast, T: new bone, M: mineralized collagen, red arrow: newly formed capillary vessel.

For the imaging of alveolar bone regeneration *in vivo*, histological analysis was performed in three rabbits implanted with MC, MC/BMP-2 and MC/BMP-2/VEGF and sacrificed at 12 weeks, respectively (Fig. 5). It was found that osteoblast-containing transplants produced abundant levels of new bone that strongly attached to the scaffold and adjacent bone (Fig. 5, C: osteoblast; T: new bone trabecular). Samples treated with MC/BMP-2 and MC/BMP-2/VEGF showed the formation of new bone that was irregular bone with a mature Haversian system. Basophilic mineralization was observed following von Kossa staining. An interface between the host and newly regenerated bone was also observed. After 12 weeks, gaps in the implants due to scaffold degradation were replaced with newly formed ingrowth bone (Fig. 5, M: materials). After 12 weeks, high levels of scaffold degradation were observed with simultaneous bone filling in the cavities, relatively mature bone and well-arranged bone trabecula and the growth of blood vessels (Fig. 5c, red arrow: newly formed capillary vessel). The most significant differences between the implants were the levels of residual scaffolds and levels of new bone, indicative of particle resorption and replacement with mineralized tissue.

**Figure 6:**
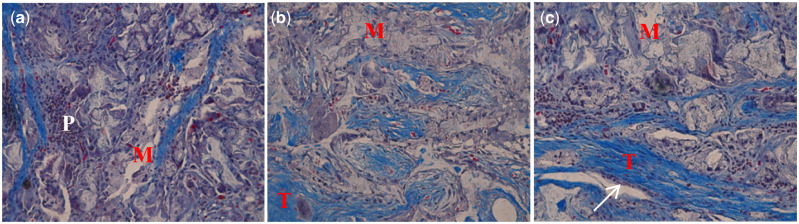
Histology photomicrographs of masson’s trichrome staining (MT ×20 magnification) of mandibular alveolar bone defects implanted with (**a**) MC, (**b**) MC/BMP2 and (**c**) MC/BMP2/VEGF at 12 weeks after surgery. P: premature bone tissue without bone cells, T: new bone, M: mineralized collagen, white arrow: lamellar bone.

Masson staining showed levels of bone formation and bone structures that were consistent with HE assessments. There were no inflammatory signs or wound healing disturbances in any of the cases. Figure 6 showed the group with BMP only or in combination with VEGF, relatively abundant new bone was observed, while the residual mineralized collagen was reduced and the appearance of higher numbers of blood vessels at the implantation site in the MC/BMP-2/VEGF group, suggestive of pro-angiogenic effects of BMP-2 by VEGF after 12 weeks of implantation. High levels of lamellar bone structure were evident in the MC/BMP-2/VEGF group. These data support the hypothesis that osteogenesis and angiogenesis are increased through the combined delivery of BMP-2 and VEGF.

### Immunohistochemistry

**Figure 7:**
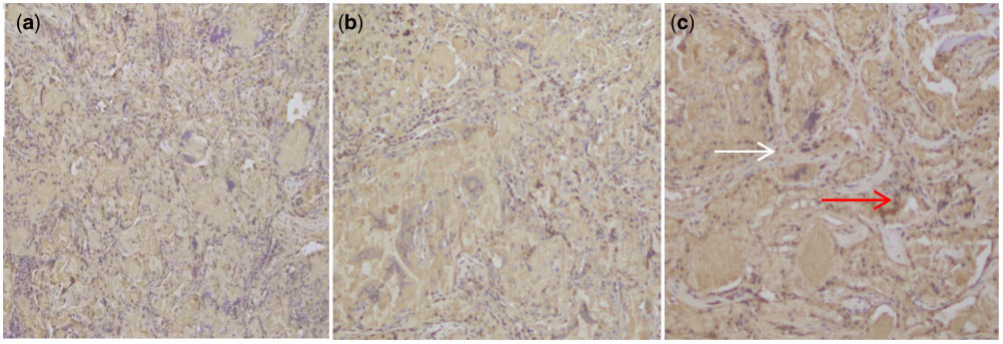
Immunochemical staining (IH ×20 magnification) OPG of mandibular alveolar bone defects implanted with (**a**) MC, (**b**) MC/BMP-2 and (**c**) MC/BMP-2/VEGF at 12 weeks after surgery. White arrow: lamellar bone, red arrow: newly formed capillary vessel.

To investigate the formation of new blood vessels around the newly formed bone, immunochemical staining OPG was performed at 12 weeks post-surgery. Areas of dense lamellar bone showed around implants with MC/BMP-2/VEGF group (Fig. 7c, white arrow). We observed higher levels of angiogenesis in the MC/BMP-2/VEGF group compared with BMP-2/MC and MC groups (Fig. 7c, red arrow). The co-delivery of BMP-2/VEGF showed dense blood vessel formation that interspersed throughout the scaffold confirming enhanced angiogenesis. These data further confirmed the utility of the BMP-2/VEGF co-delivery to improve bone regeneration and support the hypothesis that VEGF enhances bone angiogenesis to overcome osteogenic processes.

## Discussion

The utility of tissue engineering for mandibular reconstructions has been investigated in numerous animal models using a range of carriers and cell types. Biodegradable scaffolds are pivotal to bone engineering and provide skeletal support for osteogenic cell growth during the early phases of bone healing that subsequently creates gaps for new bone formation when degraded. Bone morphogenetic protein 2 (BMP-2) has been used to reconstruct mandibular defects [14]. In this study, MC/BMP-2-treated animals demonstrated significantly more bone healing than did the MC group. BMP’s osteogenic capacity is due to its ability to stimulate mesenchymal stem cells to differentiate toward an osteoblastic phenotype [16].

**Figure 8:**
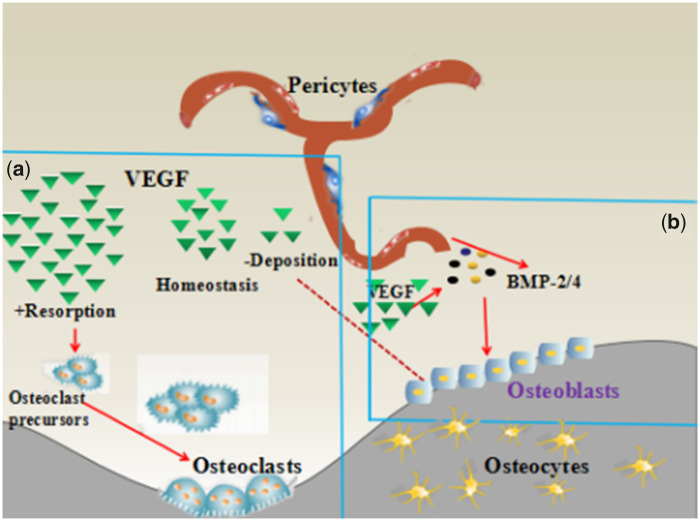
Coupling of angiogenesis and osteogenesis during intramembranous ossification. [14]

The VEGF enhances endochondral bone formation through its ability to enhance vessel invasion and chondroclast recruitment onto the hypertrophic cartilage, allowing the replacement of cartilaginous templates through the bony callus for intramembranous ossification [17–19]. In this study, MC/BMP-2/VEGF-treated animals demonstrated significantly more bone healing than did the MC group and MC/BMP-2 group. Maturing osteoblasts are a major source of VEGF, with physiological levels of VEGF maintaining bone homeostasis (as shown in Fig. 8a). Low levels of VEGF disrupt osteoblast differentiation, while its overexpression enhances osteoclast recruitment and bone resorption. Hu and Olsen showed that cortical bone defects due osteogenic cells, are key sources of VEGF. VEGF depletion disrupts angiogenic and osteogenic coupling, ultimately delaying healing [18]. VEGF regulates stem cell fate as it stimulates osteoblastic processes and inhibits adipogenic differentiation through RUNX2 and PPARy2, as opposed to paracrine signaling [20, 21]. VEGF leads to enhanced levels of BMP-2 in vessel-associated MSCs as it activates the Akt/β-catenin signaling [22]. The osteoblast mediated increase in BMP-2 acts via the autocrine system to stimulate osteoblast differentiation, stimulating VEGF production through a positive feedback loop. Figure 8b demonstrates that during bone repair, the osteoblasts produce VEGF which promotes both endothelial proliferation and migration. VEGF further upregulates BMP levels in endothelial cells. Endothelial cells secrete osteogenic factors, including BMP-2 and BMP-4, to enhance the differentiation of BMPs. Indeed, BMPs are some of the major factors controlled by VEGF in these cell types. Angiogenesis and osteogenesis are thus tightly coupled for physiological bone functions [17, 23, 24].

While these strategies offer an exciting glimpse into potential future treatments, there are several challenges that still must be overcome. Only a small amount of BMP is needed for a sizeable generation of bone, however, delivery of this bioactive molecule must be highly controlled, since overstimulation by BMP-2 has substantial side effects such as bone overgrowth and disorganization. Therefore, dose- and time-dependent effects of BMP-2 and VEGF combination on the mandibular bone generation must be highly controlled since overstimulation by growth factor has substantial side effects.

## Conclusions

In summary, we provided a novel strategy to ensure high levels of bone formation and vascularization. BMP-2 released from the MC promoted new mandibular bone formation. The combination of BMP-2 and VEGF showed a synergistic effect on mandibular bone regeneration. The direct delivery of bioactive molecules may further increase *de novo* bone formation. This knowledge provides a new therapeutic strategy to generate large-size vascularized bone grafts.

## Funding

This work was in part supported by Science Foundation of Shandong Province of China (grant no.: ZR2019PH090) and Medicine and Health Science Technology Development Plan of Shandong Province of China (grant nos: 2018WS429 and 2017WS503). 

*Conflict of interest statement.* None declared. 
