# Corrigendum to: Enhancement of BMP-2 and VEGF Carried by Mineralized Collagen for Mandibular Bone Regeneration

**DOI:** 10.1093/rb/rbaa054

**Published:** 2020-12-11

**Authors:** Kun Liu, Chun-Xiu Meng, Zhao-Yong Lv, Yu-Jue Zhang, Jun Li, Ke-Yi Li, Feng-Zhen Liu, Bin Zhang, Fu-Zhai Cui

**Affiliations:** 1 Shandong Provincial Key Laboratory of Oral Tissue Regeneration, Shandong Engineering Laboratory for Dental Materials and Oral Tissue Regeneration, School and Hospital of Stomatology, Shandong University, Jinan, Shandong 250012, P. R. China; 2 Liaocheng People’s Hospital, Medical College of Liaocheng University, Liaocheng 252000, P. R. China; 3 College of Materials Science and Engineering of Liaocheng University, Liaocheng 252000, P. R. China; 4 Department of Materials Science and Engineering, Tsinghua University, Beijing 100084, P. R. China

doi: 10.1093/rb/rbaa022, *Regenerative Biomaterials* 2020

In the originally published version of this article, the caption for Figure 8. did not credit the original source of the image: “Grosso A, Burger MG, Lunger A et al. It takes two to tango: coupling of angiogenesis and osteogenesis for bone regeneration. *Front. Bioeng. Biotechnol.* 2017; **5**:1-7.”. This error has now been corrected, and the authors apologize for this oversight.

